# Avoiding Pandemic Fears in the Subway and Conquering the Platypus

**DOI:** 10.1128/mSystems.00050-16

**Published:** 2016-06-28

**Authors:** A. Gonzalez, Y. Vázquez-Baeza, J. B. Pettengill, A. Ottesen, D. McDonald, R. Knight

**Affiliations:** a Department of Pediatrics, University of California San Diego, San Diego, California, USA; b Department of Computer Science and Engineering, University of California San Diego, San Diego, California, USA; c Food and Drug Administration, Center for Food Safety and Applied Nutrition (CFSAN), College Park, Maryland, USA; d Institute for Systems Biology, Seattle, Washington, USA

## Abstract

Metagenomics is increasingly used not just to show patterns of microbial diversity but also as a culture-independent method to detect individual organisms of intense clinical, epidemiological, conservation, forensic, or regulatory interest. A widely reported metagenomic study of the New York subway suggested that the pathogens *Yersinia pestis* and *Bacillus anthracis* were part of the “normal subway microbiome.” In their article in mSystems, Hsu and collaborators (mSystems 1(3):e00018-16, 2016, http://dx.doi.org/10.1128/mSystems.00018-16) showed that microbial communities on transit surfaces in the Boston subway system are maintained from a metapopulation of human skin commensals and environmental generalists and that reanalysis of the New York subway data with appropriate methods did not detect the pathogens. We note that commonly used software pipelines can produce results that lack *prima facie* validity (e.g., reporting widespread distribution of notorious endemic species such as the platypus or the presence of pathogens) but that appropriate use of inclusion and exclusion sets can avoid this issue.

## Abstract (precis)

Metagenomics is increasingly used not just to show patterns of microbial diversity but also as a culture-independent method to detect individual organisms of intense clinical, epidemiological, conservation, forensic, or regulatory interest. A widely reported metagenomic study of the New York subway suggested that the pathogens *Yersinia pestis* and *Bacillus anthracis* were part of the “normal subway microbiome.”

## COMMENTARY

The development and validation of novel methods that use next-generation DNA sequence data to detect pathogens from complex ecosystems represent important areas of research. In particular, these methods are important in studies of the built environment and of agricultural systems, where the correct detection of pathogens represents enormous public benefit and where incorrect detection creates fear. For example, in a recent study of the New York subway (1), due to incorrect taxonomic classifications, the authors reported observing *Yersinia pestis* (the causative agent of plague) and *Bacillus anthracis* (the causative agent of anthrax) as part of the “normal subway microbiome.” These observations led to high-visibility news reports. But improved reanalysis of the same data by Hsu et al. (2) demonstrated that these results were illusory. Hsu et al. found that these pathogens were not part of the normal subway microbiome, either in New York or in an independent sample set from the Boston subway. They drew the more plausible conclusion that the surfaces were dominated by inputs of normal human skin bacteria, consistent with other studies, and found that the subway was not a reservoir of bacterially encoded toxins or antimicrobial resistance elements. That carefully conducted study added fundamentally to our knowledge of the transmission and expression of microbes in high-traffic built environments.

Another example of the importance of accurate pathogen identification from next-generation sequencing data is the ability to detect *Salmonella* from fresh produce. In a study by Ottesen et al. (3), the authors could not confirm the presence of *Salmonella* on the tomato crops through the use of 16S amplicon sequencing. However, an analysis of shotgun data from samples collected from the roots, leaves, and fruits of the tomato plants performed using the MG-RAST server reported hits corresponding to *Salmonella*. Furthermore, this analysis also showed the surprising presence of *Gallus gallus* (red jungle fowl), *Mus musculus* (house mouse), and even the elusive *Ornithorhynchus anatinus* (duck-billed platypus).

### Detecting the presence of specific taxa from MG-RAST public datasets.

To exemplify the pervasiveness of false positives in MG-RAST, we downloaded all public samples (25,943 samples; accessed 22 April 2015), searched each report for *Salmonella*, *Raphus* (dodo bird), *Thylacinus* (Tasmanian tiger), and *Ornithorhynchus* (duck-billed platypus), and summarized the findings by the countries in which these organisms were observed on the basis of the latitude and longitude fields in the associated metadata (Table 1). A Jupyter (8) Notebook reproducing this report can be found in http://goo.gl/UIhBjf.

**TABLE 1:** Number of hits to specific taxa, living and extinct, and locations as reported by MG-RAST

Taxonomy	Extinct	Total no. of hits reported by MG-RAST	Main country locations (no. of hits [sorted by abundance])
*Ornithorhynchus*	No	17,140,078	Brazil (4,338,217), Australia (3,905,173), United States (2,669,553), Italy (2,665,186), Malawi (1,335,746), undefined (585,412), Kyrgyzstan (558,786), Russian Federation (333,978), South Africa (289,642), Belgium (198,052), Finland (168,848), China (50,542), Israel (27,366), Philippines (13,577)
*Raphus*	Yes	11	Brazil (8), Australia (3)
*Salmonella*	No	146,842,227	Italy (76,730,072), Brazil (33,956,417), United States (14,178,170), Malawi (3,808,783), China (3,383,261), Australia (3,354,697), undefined (3,257,862), Russian Federation (2,750,106), Finland (1,886,515), Belgium (1,373,668), South Africa (1,105,658), Israel (783,766), Philippines (232,026), Kyrgyzstan (41,226)
*Thylacinus*	Yes	1,344	Brazil (920), Australia (125), United States (80), Malawi (63), undefined (46), South Africa (32), Finland (23), Belgium (21), Russian Federation (15), Italy (13), Israel (4), China (2)

### Conquering the platypus.

To demonstrate how the problem of confirming the presence of specific taxa in metagenomic samples can be addressed, we created Platypus Conquistador (https://github.com/biocore/Platypus-Conquistador), a BSD-licensed Python package based on BLAST (4) and SortMeRNA (5). Platypus Conquistador confirms the presence or absence of a taxon of interest within shotgun metagenomic datasets by relying on two reference sequence databases: an inclusion database, which includes the sequences of interest (e.g., *Salmonella*), and an exclusion database, which includes any known sequence background (e.g., platypus). The reference sequence databases are expected to be mutually exclusive. In general, these two databases can be created by partitioning an existing database, such as the gene data provided by the Integrated Microbial Genomes (IMG) (6) system. These partitions can be customized to include taxa of specific interest. This method has been used by Ottesen et al. (7) to describe the efficacy of enrichment steps in the effort to culture *Salmonella* from tomatoes. For that analysis, the authors ran Platypus Conquistador on shotgun metagenomic data using the IMG database split into a reference database, including only those sequences assigned to *Salmonella*, and an exclusion database containing all remaining sequences, demonstrating the absence of this pathogen.

### Conclusions.

Simple bioinformatics solutions exist to detect taxa of interest and to resolve incorrect taxonomic classifications for shotgun sequencing data. Incorrect but pervasive taxonomic classifications can lead to conclusions that lack *prima facie* validity (for example, environments in which the platypus was reportedly found include environments from the built environment to the human gut). Worse, these incorrect assignments have great potential to spark unwarranted public concern, as was seen in the case of the NYC subway microbiome paper noted above.

These examples should also serve as a reminder that, although analytical software pipelines and computational methods can be thoroughly tested and validated, their results are based on user-specified parameters that change the results and, as a consequence, their validity. Researchers must always question the rationality of the parameters and meaning of the results to reduce the possibility of incorrect conclusions. Moving toward standardized and reproducible pipelines of analysis that can be scrutinized by our peers will greatly help avoid similar problems in the future. For pathogen detection, it is critical to additionally define taxon inclusion and exclusion criteria based on the studied environment in order to discard misleading results. This is especially important in cases of intense public interest, such as exposure in systems used by millions of people every day to apparent pathogens that are as illusory as the benthic Platypus.
